# Screening potential insect vectors in a museum biorepository reveals undiscovered diversity of plant pathogens in natural areas

**DOI:** 10.1002/ece3.7502

**Published:** 2021-05-01

**Authors:** Valeria Trivellone, Wei Wei, Luisa Filippin, Christopher H. Dietrich

**Affiliations:** ^1^ Illinois Natural History Survey Prairie Research Institute University of Illinois Champaign IL USA; ^2^ Molecular Plant Pathology Laboratory Beltsville Agricultural Research Center Agricultural Research Service United States Department of Agriculture Beltsville MD USA; ^3^ CREA–VE Council for Agricultural Research and Economics Research Centre for Viticulture and Enology Conegliano, Treviso Italy

**Keywords:** coevolution, emerging disease, leafhoppers, phytoplasma, vector‐borne pathogens

## Abstract

Phytoplasmas (*Mollicutes*, *Acholeplasmataceae*), vector‐borne obligate bacterial plant parasites, infect nearly 1,000 plant species and unknown numbers of insects, mainly leafhoppers (Hemiptera, Deltocephalinae), which play a key role in transmission and epidemiology. Although the plant–phytoplasma–insect association has been evolving for >300 million years, nearly all known phytoplasmas have been discovered as a result of the damage inflicted by phytoplasma diseases on crops. Few efforts have been made to study phytoplasmas occurring in noneconomically important plants in natural habitats. In this study, a subsample of leafhopper specimens preserved in a large museum biorepository was analyzed to unveil potential new associations. PCR screening for phytoplasmas performed on 227 phloem‐feeding leafhoppers collected worldwide from natural habitats revealed the presence of 6 different previously unknown phytoplasma strains. This indicates that museum collections of herbivorous insects represent a rich and largely untapped resource for discovery of new plant pathogens, that natural areas worldwide harbor a diverse but largely undiscovered diversity of phytoplasmas and potential insect vectors, and that independent epidemiological cycles occur in such habitats, posing a potential threat of disease spillover into agricultural systems. Larger‐scale future investigations will contribute to a better understanding of phytoplasma genetic diversity, insect host range, and insect‐borne phytoplasma transmission and provide an early warning for the emergence of new phytoplasma diseases across global agroecosystems.

## Graphical abstract

Phytoplasmas are a diverse group of obligate intracellular bacterial parasites, and diseases associated with these bacteria are among the most important problems affecting agriculture worldwide. However, most knowledge of the diversity and ecology of phytoplasmas and their hosts has been accumulated through studies of phytoplasma disease epidemiology in agroecosystems. In light of recent attention to the importance of wildlife as reservoirs of emergent diseases, we screened potential insect vectors collected in natural areas and preserved in museum biorepositories, 6 of them were positive for the presence of phytoplasmas.

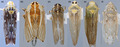

## 1 INTRODUCTION

Phytoplasmas (*Mollicutes*, *Acholeplasmatales*, *Acholeplasmataceae*) are a large group of phloem‐restricted, cell wall‐less, vector‐borne bacteria that infect hundreds of plant species and cause serious economic loss worldwide (Rao et al., 2018). In plants, phytoplasma infection may induce a variety of typical symptoms including virescence, phyllody, and witches’‐broom, thereby altering plant morphology, growth patterns, and architecture (MacLean et al., 2011, 2014; Wei et al., 2013, 2019), although infections may also be asymptomatic (Zwolinska et al., 2019).

Phytoplasmas are transmitted from plant to plant by phloem‐feeding hemipteran insect vectors, mainly leafhoppers, in a persistent‐propagative manner (Hogenhout et al., 2008; Lee et al., 2000; Weintraub & Beanland, 2006). After acquisition of phytoplasmas from an infected plant by a hemipteran insect, the phytoplasma cells must cross the midgut epithelium, then multiply in the hemolymph in order to invade the salivary glands before being inoculated into another host plant (Hogenhout et al., 2008; Huang et al., 2020; Koinuma et al., 2020).

Attempts to culture phytoplasmas in vitro have, thus far, not succeeded. Thus, phytoplasmas are currently assigned to the provisional genus “*Candidatus* (*Ca*.) Phytoplasma,” and 45 “*Ca*. Phytoplasma” species have been described (IRPCM, 2004; Kirdat et al., 2020; Naderali et al., 2017; Rodrigues Jardim et al., 2020; Šafářová et al., 2016; Zhao et al., 2021). Nevertheless, the phytoplasma lineage is a highly diverse monophyletic group (Gupta et al., 2018; Zhao et al., 2015), having been classified into 36 groups, and more than 150 subgroups based on distinct 16S rRNA gene restriction fragment length polymorphism patterns and sequencing (Lee et al., 1998; Naderali et al., 2017; Rodrigues Jardim et al., 2020; Seemüller et al., 1998; Wei et al., 2007; Zhao et al., 2009).

The intimate tritrophic interaction among phytoplasmas, host plants, and insect vectors defines a complex of multiple pathosystems worldwide (Trivellone, 2019). Almost all phytoplasma–host associations have been characterized by testing plants showing symptoms of diseases in agroecosystems. However, because the association between phytoplasmas, plants, and insect vectors has been evolving for at least 300 million years (Cao et al., 2020), phytoplasmas and their vectors should also be widespread and diverse in nonmanaged, native habitats (Trivellone & Dietrich, 2020). Indeed, current theories of infectious disease evolution suggest that most epidemic diseases afflicting humans, livestock, and crops emerge as a result of potentially pathogenic organisms “jumping” from a native host to a new host following anthropogenic disturbance of natural habitats (Brooks et al., 2019).

About 100 insect species have been recorded as competent vectors of phytoplasmas; however, for most the of described “*Ca*. Phytoplasma” species and 16S rRNA subgroups the suite of vectors is still unknown (overview in Trivellone, 2019). Because insects are often difficult to identify and individuals infected with phytoplasmas cannot be distinguished from noninfected individuals except through microscopy, molecular screening, or pathogen transmission trials, efforts to identify competent phytoplasma vectors have lagged far behind efforts to characterize phytoplasmas and their host plants. Due to the mobility of insect vectors, spillovers of vector‐borne phytoplasmas from adjacent highly diverse natural habitats into agroecosystems were hypothesized to play an important role in emergence of new phytoplasma diseases (see Brooks et al., 2021). However, few attempts have been made to study phytoplasma diversity in natural habitats. Therefore, diversity, plant host range, and insect vector range of phytoplasmas are probably significantly underestimated (Trivellone & Dietrich, 2020).

Due to increased awareness of the importance of wildlife as pathogen reservoirs (Brooks et al., 2020), the use of museum biorepositories to discover and track pathogens is a critical step for anticipating the emergence and re‐emergence of infectious diseases (DiEuliis et al., 2016; Dunnum et al., 2017). The high levels of biodiversity and geographic coverage represented in such repositories can also help unveil the evolutionary history of pathogens and reveal previously unknown interactions with actual or potential hosts.

In this study, we analyzed specimens of Deltocephalinae leafhoppers (Hemiptera: Cicadellidae: Deltocephalinae) preserved in the collection of the Illinois Natural History Survey (INHS) (http://inhsinsectcollection.speciesfile.org/InsectCollection.aspx). The INHS leafhopper collection is one of the largest in world with over > 400,000 specimens stored either pinned or in ethanol at −20°C. In 2018, a subsample of ethanol‐preserved leafhoppers collected in natural habitats were tested for the presence of phytoplasmas. The results revealed that about 3% of tested insect specimens harbored phytoplasmas. The newly discovered phytoplasmas group with phytoplasma strains belonging to three distinct taxonomic (16Sr) groups. Phytoplasmas were detected from a total of six leafhopper species including five known and one recently described species, all recorded for the first time as potential phytoplasma vectors. These results indicated that phytoplasma diversity and potential insect host range are indeed underestimated, and further large‐scale investigation of leafhopper samples collected from natural habitats is needed.

## 2 MATERIALS AND METHODS

### 2.1 Collection and preservation of leafhoppers

**FIGURE 1:**
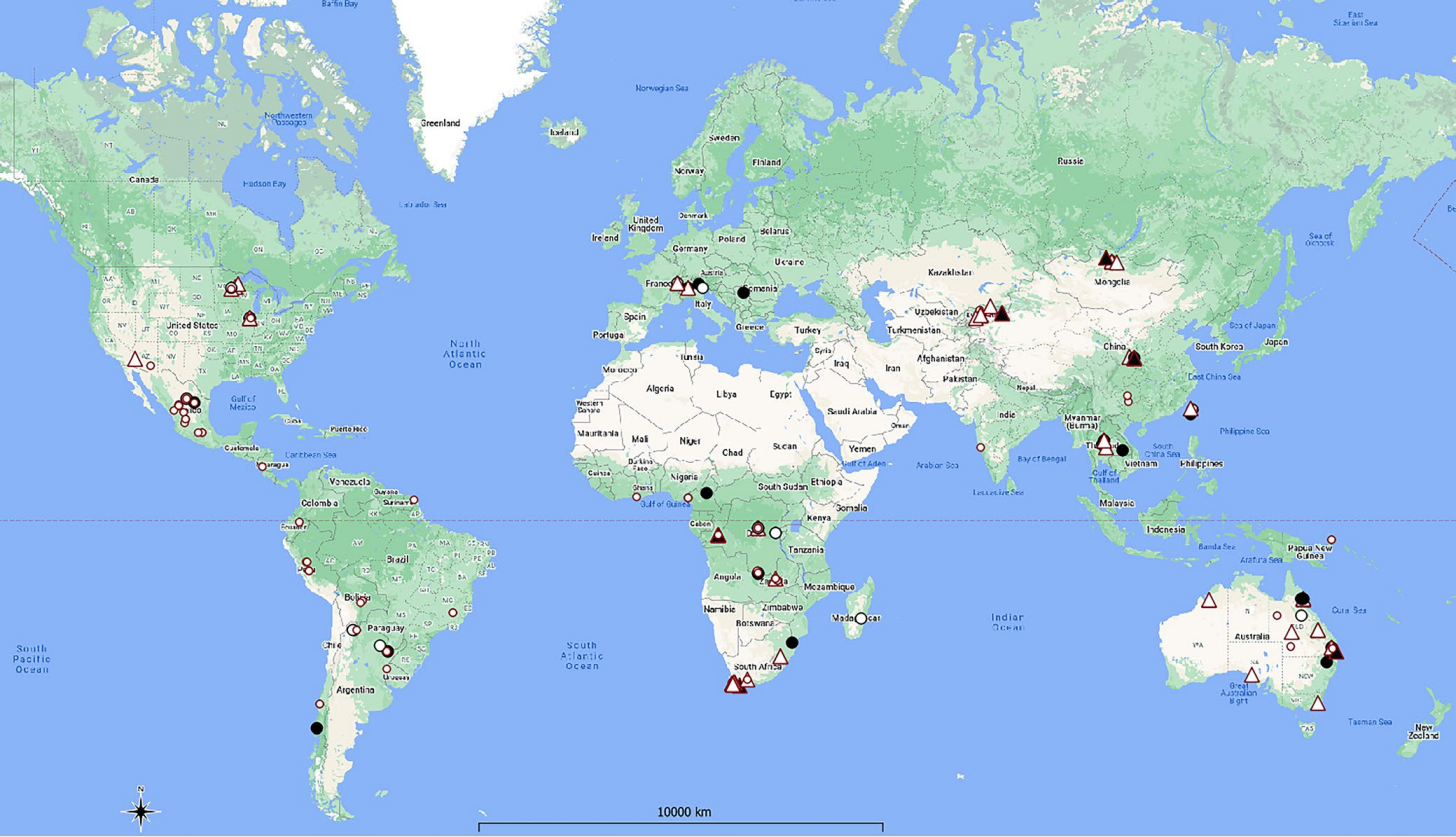
Map of the sampling sites of the 227 leafhopper samples screened in the present study. Symbols indicating the Cq value results of qPCR (small empty circle: negative; big empty circle: Cq > 41; big black circle: 35.54 < Cq ≤ 40; empty triangle: 31 < Cq < 35.52; black triangle: Cq ≤ 30.38). Only black triangle symbols are considered phytoplasma‐positive samples and further analyzed with nested PCR. Map created QGIS 3.8 and was modified with Adobe Photoshop CC 2019. This map is licensed under an X/MIT style Open Source License by the Open Source Geospatial Foundation

More than 3,000 bulk samples of sap‐feeding hemipteran insects were obtained between 1998 and 2018 through fieldwork by the last author, his students, and colleagues during surveys that aimed to document poorly studied insect faunas in various parts of the world and to obtain representatives of all major lineages of Cicadellidae for use in phylogenetic and systematic studies. This material was supplemented by the first author's collections in Europe between 2001 and 2018. Specimens were collected using various methods including sweeping and vacuuming of vegetation, night collecting at lights, and in Malaise (flight intercept) traps. Specimens were collected directly into 95% ethanol in the field, returned to the laboratory and stored in −20°C freezers at the INHS. Voucher specimens were also pinned for species identification and reference. Some samples included undescribed species from under‐investigated areas, and they are waiting to be described in the context of other projects. In 2018, screening was carried out on a subset of 227 samples from independent sampling events in 28 countries (six continents) worldwide (Argentina, Australia, Bolivia, Brazil, Cameroon, Chile, China, Czech Republic, Ecuador, French Guiana, Ghana, Kyrgyzstan, India, Italy, Madagascar, Mongolia, Nicaragua, Papua New Guinea, Peru, Republic of Congo, Serbia, South Africa, Swaziland, Switzerland, Taiwan, Thailand, the United States, and Zambia). The land cover of the sampling events was analyzed using thematic maps within a geographic information system (QGIS3.8, 2019; Figure 1). Although 98% of the collections were intentionally obtained from natural areas or patches of native vegetation within more anthropogenic landscapes, we evaluated the land cover of a larger area including each sampling site using the raster layer Cropland and Pasture area (resolution 10 × 10 km; Ramankutty et al., 2008).

In total, the 227 samples encompassed about 1,000 specimens, with each species (or morphospecies) represented by 1 to 20 specimens belonging to the phloem‐feeding leafhopper subfamily Deltocephalinae (except 1 sample belonging to the related hemipteran family Membracidae), which includes most of the previously documented vectors of phytoplasmas (Table S1). At least one specimen from each sample was selected randomly (with preference for males when present because species identification usually requires examination of male genitalia) for the molecular analyses.

### 2.2 DNA extraction

Total DNA was extracted from individual leafhoppers using a nondestructive method to preserve the specimen exoskeletons as vouchers and for subsequent morphological study. For each specimen, the abdomen was dissected, transferred to a 1.5‐ml tube containing 400 µl 1X TES pH 7.8 buffer (20 mM Tris, 10 mM EDTA, 0.5% SDS) and 4 µl Proteinase K (20 mg/µl), and incubated at 56°C overnight. The abdomen was then removed and preserved in ethanol for morphological study. The buffer solution was then blended for 10 min using a mixer (MixMate), and the solution was transferred to a new 1.5‐ml tube with 400 µl of chloroform, mixed, and centrifuged for 10 min at 4°C at 13552 RCF. The supernatant was transferred to a new tube, and the chloroform wash was repeated. DNA was then transferred to a new tube, and 400 µl of ice‐cold isopropanol was added followed by mixing and centrifuging for 15 min at 4°C at 16128 RCF. Supernatant was discarded, and the DNA pellet was washed twice using 500 µl of ice‐cold 96% ethanol. The DNA pellet was then dried for 20 min and resuspended in 50 µl of TE buffer (pH 7.8). To each leafhopper sample, a molecular code was assigned: For example, LH078 stands for LeafHopper followed by an ordinal number indicating the collection event.

### 2.3 Leafhopper species identification

Specimens were sorted to morphospecies and tentatively identified by the last author prior to DNA extraction, with species identifications confirmed following nondestructive DNA extraction through examination of male genitalia. Exoskeletons of extracted specimens were saved as vouchers and deposited in the Illinois Natural History Survey insect collection. After the initial screening, all the specimens that tested positive for the presence of phytoplasmas were identified by using published taxonomic keys and related literature (Emeljanov, 1967; Fletcher, 2000; Stiller, 2010; Zahniser, 2008). One of them was a new species for science and was recently described by the last author (Dietrich, 2021). The abdomens of voucher specimens (males) were dissected to study the genitalia under an Olympus SZX10 stereoscopic microscope. Habitus photographs of voucher specimens were taken at INHS with a Canon SLR camera and 65‐mm macro lens mounted on an automated lift.

### 2.4 DNA amplification and sequencing of phytoplasmas

TaqMan real‐time PCR (qPCR) analysis of the 16S ribosomal gene was carried out on DNA extracted individually from the 227 specimens to identify the presence of phytoplasmas, with the primers and probe described by Christensen et al. (2004). The assays were performed in 96‐well plates on a CFX96 thermal cycler (Bio‐Rad), according to the protocol of Angelini et al. (2007). The reaction in 10 µl contained 4 µl of DNA template diluted 1:2, 5 µl Platinum Quantitative PCR Supermix‐UDG (Thermo Fisher Scientific), 160 nM for each primer and probe. Because this protocol may yield false positives for other bacteria (e.g., *Bacillus* spp.), samples with Cq value ≤ 30.38 (based on results in Christensen et al., 2004) were tested using nested PCR of the 16S ribosomal RNA gene to confirm the phytoplasma identity. In the 16S rRNA region, nested PCR was performed using universal primer pair P1/P7 (Deng & Hiruki, 1991; Smart et al., 1996) followed by F2n/R2 (Gundersen & Lee, 1996). Amplicons were visualized on 1% agarose gel stained with GelRed (Biotium Inc.) under a Gel Doc XR UV transilluminator (Bio‐Rad). The DNA of ALY (Italian alder yellows) phytoplasma, obtained from experimentally infected periwinkle (*Catharanthus roseus*), was used as a positive reference strain in all the amplification reactions. Sequencing of the F2n/R2 amplicons was carried out in both directions using automated equipment (BMR Service, Padua, Italy). Forward and reverse reads were assembled using Gap4 and Pregap (Bonfield et al., 1995), followed by manual editing. Nucleotide sequences were deposited in the GenBank database under the accession numbers listed in Table 1. An initial BLAST query (Altschul et al., 1990) was performed in order to evaluate the similarity of newly obtained sequences to the five most similar phytoplasma sequences evaluated for inclusion (Table S2) in the final dataset for further phylogenetic analyses. The final reference sequence dataset consisted of 21 sequences obtained from the National Center for Biotechnology Information (NCBI) database (Federhen, 2012). The ingroup included 20 phytoplasma strains (11 described as “*Ca*. Phytoplasma” species, including an incidental citation) representing different countries and isolated from distantly related hosts (Table S3), and the outgroup included *Acholeplasma palmae* (*Acholeplasmataceae*). Electropherograms were corrected and aligned using the Muscle algorithm as implemented in MEGA 7.0 (Edgar, 2004; Kumar et al., 2016) with default settings. The final aligned dataset is available at https://doi.org/10.13012/B2IDB‐2694515_V1. Phylogenetic trees were constructed with the maximum‐likelihood (ML) method based on the Kimura 2‐parameter model (Kimura, 1980) and neighbor‐joining (NJ) method using the maximum composite likelihood model (Tamura et al., 2004). Branch support was measured using a bootstrap test with 1,000 replicates.

**TABLE 1:** List of species collected in natural areas that tested positive for the presence of phytoplasmas. Description of locations of the new associations between phytoplasmas and insect hosts detected in this study

Code	Tribe	Species	Country	Coordinate	Habitat, altitude (m a.s.l.)	Date Collection method Collector	Phytoplasma^a^ (Accession number)
LH78	Chiasmini	*Leofa dispar*	South Africa	28°53′59″S 29°26′05″E	Grassland, 1,583	27 Dec 2004 Sweep net J.N. Zahniser	16Sr XI/XIV (MW473669)
LH82	Paralimnini	*Pravistylus exquadratus*	South Africa	33°51′01″S 19°03′16″E	Fynbos, 201	15 Dec 2004 Vacuum J.N. Zahniser	16Sr XI/XIV (MW473673)
LH102	Macrostelini	*Macrosteles sordidipennis*	Kyrgyzstan	41°47′52″N 78°39′44″E	Sedge meadow 2,950	04 Jul 1999 Vacuum D. Novikov & C.H. Dietrich	16Sr I (MW473674)
LH133	Paralimnini	*Mayawa capitata*	Australia	32°57′06″S 115°54′49″E	Yarloop Nature Reserve, 76	10 Jan 2010 Sweep net K. Hill, et. al.	16Sr II/XV (MW473671)
LH139	Paralimnini	*Mayawa affinifacialis*	Australia	27°56′03″S 153°04′42″E	Flagstone Creek Reserve Park, on grassland, 50	04 Jan 2009 Sweep net K. Hill, et. al.	16Sr XIV (MW473672)
LH143	Paralimnini	*Acharis ussuriensis*	China	33°58′53″N 108°09′50″E	Forest Natural Reserve, 660	12 July 2012 Vacuum Wei Cong	16Sr XI/XIV (MW473670)

^a^ The last column indicates the 16Sr phytoplasma group(s) of the strains most similar to the new strains according to the ML phylogenetic analysis of 16S rRNA gene and the GenBank accession numbers of the deposited sequences.

## 3 RESULTS

### 3.1 Taxonomic diversity of tested leafhopper samples

The 227 specimens analyzed belong to 9 tribes (Athysanini, Chiasmini, Deltocephalini, Macrostelini, Opsiini, Paralimnini, Pendarini, Scaphoideini, and Scaphytopiini), which represent most of the groups of Deltocephalinae comprising known phytoplasma vectors worldwide. Overall, about 49% of them (111 specimens) were identified to species during earlier sorting and preparation of collected samples, 2% are of uncertain species placement, and ~43% represent undescribed species and genera or belong to genera for which comprehensive identification tools are not yet available. Thirteen species (6%) are represented by multiple specimens (Table S1).

GIS analyses with the Cropland and Pasture overlay confirmed that the sampling sites were located mainly in natural areas, with average raster values of 0.091 ± 0.13 (compared with cropland raster value = 1).

### 3.2 Detection and phylogenetic analysis of phytoplasmas

**FIGURE 2:**
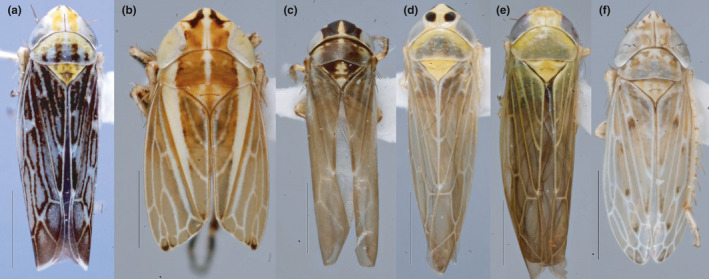
Dorsal views of the 6 species of leafhoppers that tested positive for phytoplasmas. a, *Acharis ussuriensis* (Melichar) (molecular code, LH143); b, *Leofa* (*Tortotettix*) *dispar* (Theron) (LH078); c, *Macrosteles* (*Macrosteles*) *sordidipennis* (Stål) (LH102); d, *Mayawa capitata* (Kirkaldy) (LH133); E, *Mayawa affinifacialis* Dietrich (LH139); F, *Pravistylus exquadratus* (Naudé) (LH082). Scale bar 1.0 mm

Using qPCR on 227 leafhoppers, a positive signal was detected in 111 specimens. Only 14 samples with Cq value ≤ 30.38 were selected for further analysis (Table S1). The nested PCR primed by F2n/R2 amplified fragments of the 16S rRNA gene of 1,200 bp from 6 phytoplasma‐infected samples (Fig. S1). A lower or different sensitivity of the direct/nested primers compared with the ones used in qPCR may have caused the negative results for the remaining 8 samples. Overall, 6 species tested positive: *Leofa* (*Tortotettix*) *dispar* (Theron) (molecular sample codes: LH078), *Pravistylus exquadratus* (Naudé) (LH082), *Macrosteles* (*Macrosteles*) *sordidipennis* (Stål) (LH102), *Mayawa capitata* (Kirkaldy) (LH133), *Mayawa affinifacialis* Dietrich (LH139), and *Acharis ussuriensis* (Melichar) (LH143) (Table 1, Figure 2a‐f).

**FIGURE 3:**
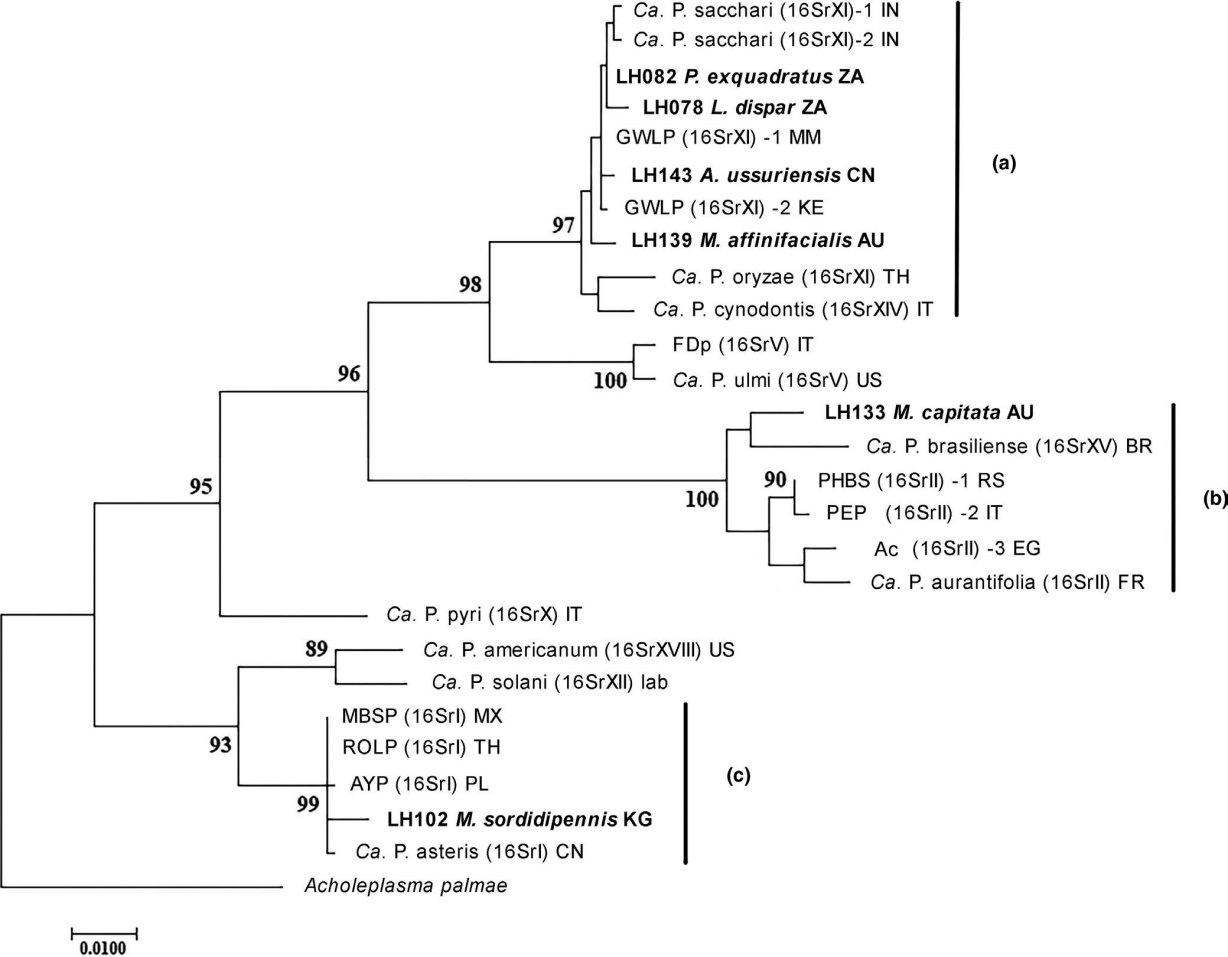
Maximum‐likelihood tree based on 952 positions of the F2n/R2 fragment of the *16S rRNA* gene obtained from 6 samples of the present study (in bold), 20 phytoplasma strains from GenBank (used as references) and *Acholeplasma palmae* (outgroup). Bootstrap values (> 63%) are shown above or below the branches. Branch lengths are proportional based on the scale indicated. Initial tree(s) for the heuristic search were obtained automatically by applying the Maximum Parsimony method. GenBank accession numbers and details of the reference phytoplasma strains are listed in Table S3. The names at the tip of the tree include the following: the phytoplasma strain (acronym or *Candidatus* species name), the 16Sr phytoplasma group in parenthesis or the name of the insect species host, and the Country Code where the strain was detected (AU, Australia; BR, Brazil; CN, China; EG, Egypt; FR, France; IT, Italy; IN, India; KE, Kenya; KG, Kyrgyzstan; MM, Myanmar; MX, Mexico; PL, Poland; RS, Serbia; TH, Thailand; US, United States; ZA, South Africa). A, B, and C indicate the clusters that include the samples from this study

The phylogenetic trees included 27 phytoplasma strains, and the alignment of 16S rRNA consisted of 952 positions (including gaps). Trees obtained from ML and NJ analysis were well‐resolved and identical. Although the number of included strains is much lower in our dataset, the topology obtained is congruent with that of the recent comprehensive phylogenetic analysis of Cao et al. (2020). The phylogeny placed our new sequences in three main clusters (A, B, and C in Figure 3). The first well‐supported cluster (A) includes a monophyletic group of 4 samples from this study (LH078, LH082, LH139, and LH143) and 4 strains belonging to 16SrXI phytoplasma group (2 “*Ca*. Phytoplasma sacchari” and 2 Goosegrass white leaf phytoplasma strains) and the *Candidatus* species “*Ca*. Phytoplasma oryzae” (16SrXI) + “*Ca*. Phytoplasma cynodontis” (16SrXIV). Although the internal branches of this clade are very short with low bootstrap support, both samples from South Africa (LH078, LH082) were recovered in the same subcluster. *Leofa dispar* (LH078) and *P. exquadratus* (LH082) were collected from native grassland and fynbos vegetation in two different provinces in South Africa (KwaZulu‐Natal and Western Cape) in 2004 (Table 1). The distance between these two sampling sites is about 1,120 km.

Samples from China (LH143) and Australia (LH139) are polyphyletic, with LH139 branching more deeply than LH143. A recent comprehensive ML tree for phytoplasmas recovered members of 16SrXI as paraphyletic with respect to 16SrXIV (Cao et al., 2020). Both *Acharis ussuriensis* (LH143) and *M. affinifacialis* (LH139) were collected from nature reserves. *Acharis ussuriensis* was collected in China from grasses on a dry hillside at Zhouzhi Nature Reserve (Zhouzhi county, Shaanxi Province). The sampling location is entirely surrounded by forest with the nearest farming settlement about 10 km away. *M. affinifacialis* was collected in Australia in Yarloop Nature Reserve (Figure 1 and Table 1).

In the second cluster (B), LH133 is sister to “*Ca*. Phytoplasma brasiliense” (16SrXV), and together, these two strains are sister to the closely related phytoplasma strains in the 16SrII group. *Mayawa capitata* (LH133) was collected at lights from a nature reserves in Australia, and the sampling site is about 3,597 km away from the site where *M. affinifacialis* was collected (Figure 1).

The last cluster (C) includes LH102 and members of the 16SrI phytoplasma group. The *Macrosteles sordidipennis* (LH102) specimen and other individuals were collected in a riparian sedge meadow, on a river bank in Kyrgyzstan (Jeti‐Ögüz District) in 1999 (Figure 1). The NJ analysis yielded the same topology (not shown).

## 4 DISCUSSION

### 4.1 Ecological and evolutionary context of the new phytoplasma–host associations

None of the leafhopper species that tested positive for the presence of phytoplasmas in the present study were previously reported as hosts or vectors of phytoplasmas (Trivellone, 2019). These 6 leafhopper specimens were collected from native grassy vegetation in four countries: South Africa, Kyrgyzstan, Australia, and China (Table 1 and Figure 1). In South Africa, only four 16Sr phytoplasma groups were previously recorded (16SrI, 16SrII, 16SrIII, and 16SrXII) (for an overview, see Trivellone, 2019), and only two species of leafhoppers were recorded as potential vectors and competent vectors of phytoplasmas in the 16SrI group: *Austroagallia* sp. (subfamily Megophthalminae) and *Mgenia fuscovaria* (Stål) (Coelidiinae) (Kruger et al., 2015). Thus, this is the first record of phytoplasma strains in the clade 16SrXI/16SrXIV in South Africa. The leafhopper fauna of Africa is diverse but remains poorly known, with new genera and species continuing to be discovered (e.g., Stiller, 2019, 2020). *Pravistylus exquadratus* and other members of the same genus have never been reported as pests, except for single records of this species on Korog wheat cultivar and on ryegrass (Stiller, 2010). The species is mainly associated with native grassland and fynbos vegetation, and it is always macropterous with high potential for dispersal. *Leofa dispar* also occurs in native grassland and has not been reported from crops. Both *P. exquadratus* and *L. dispar* are restricted to South Africa.

The single tested specimen of *Macrosteles sordidipennis* LH102 collected in Kyrgyzstan was infected by a strain of group 16SrI related to aster yellows phytoplasma strains. The only previous phytoplasma record from this country was the potato stolbur disease, associated with the 16SrXII phytoplasma group, but this was never confirmed using molecular methods. Interestingly, other species in the genus *Macrosteles* have been reported as competent vectors of phytoplasmas (Trivellone, 2019). In particular, competent and potential vectors of *Macrosteles* show a strong cophylogenetic signal with the 16SrI phytoplasma group (Trivellone, unpublished data), suggesting that these two lineages have been associated for a long time. Nine additional species of *Macrosteles* have been documented in Kyrgyzstan (Novikov et al., 2006), including four that are competent vectors of 16SrI phytoplasmas in Europe although 16SrI phytoplasmas have not been previously recorded from this country (Trivellone, 2018, 2019). Our discovery of a new association between a *Macrosteles* species not previously recorded as a phytoplasma host and a new 16SrI group strain or host suggests that further surveys and phytoplasma screening in Kyrgyzstan may be important for assessing the potential threat of emerging phytoplasma diseases in this region of Central Asia.

Among the species collected in Australia, *Mayawa capitata* (LH133) belongs to the grass‐specialist leafhopper tribe Paralimnini and reportedly occurs on grasses and *Sida acuta* (Malvaceae) (Fletcher, 2000). *Mayawa affinifacialis* (LH139) has been recently described (Dietrich, 2021), and little is known about its ecology; however, the species that was collected in grassland is likely a grass feeder. A specimen of the first species (LH133) was infected with a phytoplasma strain closely related to strains classified in the 16SrXV group and the second one (LH139) with a phytoplasma strain closely related to strains classified in the group 16SrXI. Only 3 competent vectors for phytoplasmas (all in group 16SrII) were previously known for this country, two species of *Orosius,* tribe Opsiini (Deltocephalinae), and *Batracomorphus angustatus* (Osborn) in the subfamily Iassinae (for an overview, see Trivellone, 2019). A recent review of Australian phytoplasma pathosystems revealed an important gap of knowledge, with several recorded phytoplasma strains not yet assigned to 16Sr groups and subgroups (Liu et al., 2017). Moreover, information on competent vectors is scarce with many species still undescribed, hampering the understanding of epidemiological cycles. Our results expand the spectrum of potential vectors recorded in Australia to include species from the tribe Paralimnini, and reveal new possible epidemiological routes that require further investigation.

The specimen of *Acharis ussuriensis* (LH143) testing positive was infected with a strain closely related to strains in the 16SrXI/16SrXIV groups (Fig. 3, cluster A). Although both phytoplasma groups were previously detected in China, further investigation on the pattern of transmission and host plants involved in this pristine area will provide useful insights into the characterization of phytoplasma–host relationships in natural areas.

### 4.2 Underestimated phytoplasma diversity in natural areas

Phytoplasmas are a highly diverse group of plant pathogens, and new strains continue to be discovered at a steady pace worldwide. Most such discoveries still mainly result from screening of plants showing “typical” phytoplasma disease symptoms in human‐managed ecosystems.

By screening leafhopper specimens from natural habitats, we revealed new associations between phytoplasmas and their insect hosts, recording new phytoplasma group records for 3 countries. The phytoplasma strains newly detected here have been further characterized, which represent multiple subgroup lineages (2021). Our results highlight the fact that potential vectors in natural areas are poorly studied (as suggested by Trivellone & Dietrich, 2020) and may harbor phytoplasma species not yet discovered and described. Discovery of new phytoplasmas in natural areas worldwide is not surprising, given the > 300‐million‐year history of coevolution between phytoplasmas, their plant hosts, and insect vectors and the lack of extensive screening for phytoplasmas in nonmanaged ecosystems (Cao et al., 2020; Trivellone & Dietrich, 2020). According to a recent molecular timetree (Cao et al., 2020), the earliest divergences of phytoplasmas approximately coincided with those of their vascular plant hosts and some phytoplasma lineages are associated with particular major lineages of plants and hemipteran insects. If many such associations are evolutionarily conservative, then phylogenies may be useful tools for predicting undocumented associations between phytoplasmas, insects and plants. Also, because coevolutionary theory suggests that associations between parasites and their hosts should evolve toward commensalism over time (i.e., virulence should decrease; Alizon et al., 2009; Jansen et al., 2015), plants naturally infected by phytoplasmas in natural areas may not exhibit the classical symptoms of phytoplasma disease found in crop plants. Thus, many naturally occurring plant–phytoplasma associations may be asymptomatic. Screening of potential vectors and/or asymptomatic plants may be necessary to reveal the true diversity of unknown phytoplasma strains in native ecosystems. No evidence of diseased plant hosts was reported from the investigated sites where leafhoppers included in our study were collected. However, the collections were originally made for the purpose of documenting insect biodiversity, rather than within the context of plant pathogen surveys. For this reason, we cannot speculate on the disease epidemiology of phytoplasmas associated with leafhoppers tested for the present study. Further investigations are needed to document the host plants and phenotypic effects of phytoplasma infections for the newly documented strains.

Although 5 leafhopper genera recorded here as phytoplasma hosts have not been previously reported as potential or competent vectors of phytoplasmas, all belong to tribes that include known vectors. Thus, vector competence may be a phylogenetically conservative trait in some lineages of leafhoppers. Further, studies using cophylogenetic methods may be useful for predicting new pathogen–host associations and emerging diseases (reviewed in Brooks et al., 2019).

Given that most previous research on phytoplasmas has been performed within the relatively narrow context of plant disease epidemiology in agroecosystems, we suggest that the diversity of phytoplasmas is severely underestimated and that natural areas worldwide should harbor a rich undiscovered diversity of phytoplasmas and their actual or potential insect vectors.

Similar phytoplasma infection prevalence in agroecosystems and natural grassland was previously reported in the literature; however, knowledge of the entire range of hosts (plants and insects) and symptoms caused by phytoplasmas in natural habitats remains inadequate (for a review, see Trivellone & Dietrich, 2020).

### 4.3 Museum biorepository as source of unknown phytoplasmas

Previous research showed that integrating different sources of knowledge is of paramount importance for discovering potentially emergent pathogens. Studies on zoonotic diseases showed that museum biorepositories represent an invaluable but still poorly utilized resource for pathogen discovery, due to the wealth of species represented and prevalent best practices of specimen preservation, identification, and collecting event description (Dunnum et al., 2017). Furthermore, existing databases and traditional ecological knowledge can contribute to discovery of the location and timing of potential spillover of pathogens into human‐managed systems worldwide (Brook et al., 2009; Kutz et al., 2009). Plant, fungal, and animal specimens deposited in natural history museums and public or private collections are becoming increasingly accessible due to Web‐based interfaces. These collections represent the most comprehensive available sources of data documenting the diversity of life and have proven useful for many purposes beyond their traditional applications to comparative morphology, taxonomy, and biogeography (Meineke et al., 2019). Until recently, species interactions documented by collections were mainly investigated using metadata (e.g., Bartomeus et al., 2019; Meineke & Davies, 2019). The advent of increasingly sensitive molecular methods has recently allowed more cryptic symbiotic associations to be explored directly by the testing preserved tissues of potential hosts for the presence of microbes and other symbionts (e.g., Daru et al., 2019). To our knowledge, this is the first time that phytoplasma–insect associations have been documented using museum specimens. However, because most collections of leafhoppers and other terrestrial insects consist of dried, pinned specimens, the ethanol‐preserved specimens screened for our study are not typical of the material usually available in museums. Nevertheless, dried, pinned insect specimens have been shown to yield DNA of sufficient quality for use in various applications, including DNA barcoding and phylogenetics (e.g., Blaimer et al., 2016; Mitchell, 2015). We did not attempt to screen such specimens for phytoplasmas in our study. The ethanol‐preserved specimens tested for this study ranged in age from 1 to 20 years, and we detected no correlation between the quantity or quality of DNA obtained and the age of the specimen.

Our screening confirmed the presence of phytoplasmas in 6 leafhopper specimens (accounting for ~3% of the subset of 227 leafhoppers analyzed). Because we mostly tested single specimens from collecting events spread over 20 years on multiple continents, it is not surprising that most of our samples tested negative for the presence of phytoplasmas. Our data do not allow us to speculate on local infection rates of the new strains detected. However, considering the spatial, temporal, and taxonomic scale of the samples available in museum biorepositories, our results can be taken as a very rough, preliminary estimate of phytoplasma prevalence in natural areas worldwide and suggest that the undiscovered diversity of phytoplasmas in natural areas worldwide is substantial.

Given the success of our approach, larger‐scale studies of museum biorepositories have strong potential to fill major gaps in our knowledge of phytoplasma diversity, the evolution of phytoplasma–plant–vector associations, and the potential for emergence of new pathogens of agricultural importance.

### 4.4 Potential impact of vector‐borne phytoplasma spillovers and large‐scale future study

Centuries of homogenization of agricultural production systems led to decreased genetic and species diversity of crops. Such general biological depletion was previously associated with increased pathogen outbreaks and serious economic losses in agroecosystems (King & Lively, 2012; Newton, 2016). Earlier research recognized the role of wildlife as natural reservoirs where infections are often asymptomatic. The onslaught of emerging infectious diseases in crops often involved alternative sources of inoculum and creation of new ecological interfaces, and global changes (e.g., land use or climate warming) set the stage for new associations to occur. Spillover events from natural habitats in direct contact with cultivated fields have been documented for several plant pathogens (Brooks et al., 2021; McCann, 2020), and the involvement of vectors may facilitate host shifts, accelerating the spread of diseases at the regional level. The phytoplasmas associated with Flavescence dorée disease, and related strains (FDp), represent one of the most well‐studied pathosystems (Malembic‐Maher et al., 2020), providing a good example of spillover from wild plants to a crop (*Vitis vinifera*) through efficient insect vectors (Brooks et al., 2021; Trivellone & Dietrich, 2020). For other phytoplasma pathosystems, epidemiological information and characterization of strains associated with crops have accumulated for over forty years. However, information on genetic diversity, the range of hosts, and ecological characteristics of the spreading of phytoplasmas in natural habitats are still broadly missing. This gap of knowledge hinders basic understanding of the evolution of phytoplasmas in association with their hosts, and hampers the implementation of proactive measures to cope with emerging pathogens.

## CONFLICT OF INTEREST

None declared.

## AUTHOR CONTRIBUTION

**Valeria Trivellone:** Conceptualization (equal); Data curation (equal); Formal analysis (equal); Funding acquisition (equal); Methodology (equal); Writing‐original draft (equal); Writing‐review & editing (equal). **Wei Wei:** Formal analysis (supporting); Writing‐original draft (equal); Writing‐review & editing (equal). **Luisa Filippin:** Formal analysis (equal); Methodology (equal); Writing‐review & editing (equal). **Christopher H. Dietrich:** Conceptualization (equal); Funding acquisition (equal); Methodology (equal); Supervision (equal); Writing‐original draft (equal); Writing‐review & editing (equal).

## Supporting information

Fig S1

Table S1

Table S2

Table S3

## Data Availability

The sequences supporting the conclusions of this article were deposited into the GenBank under the accession numbers MW473669‐MW473674.
