# Supplementary material for: Screening potential insect vectors in a museum biorepository reveals undiscovered diversity of plant pathogens in natural areas
Source: Ecol Evol. 2021 May 1;11(11):6493–503. doi: 10.1002/ece3.7502 (PMC8207438; doi:10.1002/ece3.7502)
Supplement: Supplementary file 1 — Fig S1 [file ECE3-11-6493-s003.docx]

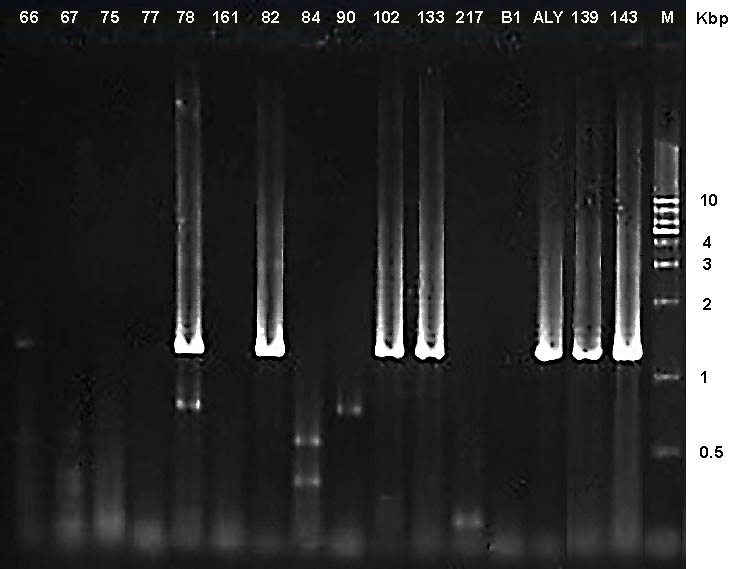


Figure S1. Agarose gel (1%) showing F2n/R2 amplicons obtained in nested PCR for 14 leafhopper samples of study with a Cq ≤30.38 in qPCR. B1: blanks, negative control; ALY, alder yellow phytoplasma; M: marker of molecular weight, 1 kb DNA ladder (RBC Bioscience). Numbers refer to the leafhopper IDs in Table S1.
